# Aberrant SUMOylation Restricts the Targetable Cancer Immunopeptidome

**DOI:** 10.1002/advs.202511449

**Published:** 2026-01-21

**Authors:** Uta M. Demel, Anna Meurer, Badeel Kh. Q. Zaghla, Bilge Atay, Daniel Steiert, Luca V. Hummel, Konstandina Isaakidis, Chuanbing Zang, Michael Korenkov, Marlon Schielin, Schayan Yousefian, Shima Mecklenbräuker, Marieluise Kirchner, Simon Haas, Antonia Busse, Philipp Mertins, Stefan Müller, Matthias Wirth, Martin G. Klatt, Ulrich Keller

**Affiliations:** ^1^ Department of Hematology, Oncology and Cancer Immunology, Charité – Universitätsmedizin Berlin Corporate Member of Freie Universität Berlin and Humboldt‐Universität zu Berlin Berlin Germany; ^2^ Max‐Delbrück‐Center For Molecular Medicine Berlin Germany; ^3^ Clinician Scientist Program Berlin Institute of Health (BIH) Berlin Germany; ^4^ Berlin Institute for Medical Systems Biology Max Delbrück Center For Molecular Medicine in the Helmholtz Association Berlin Germany; ^5^ Berlin Institute of Health (BIH) At Charité – Universitätsmedizin Berlin Berlin Germany; ^6^ German Cancer Consortium (DKTK) partner site Berlin German Cancer Research Center (DKFZ) Heidelberg Germany; ^7^ Cluster of Excellence Immunoprecept Charité – Universitätsmedizin Berlin Berlin Germany; ^8^ Precision Healthcare University Research Institute Queen Mary University of London London UK; ^9^ Institute of Biochemistry II, Goethe University Frankfurt Medical School Frankfurt Germany; ^10^ German Cancer Consortium (DKTK) partner site Frankfurt/ Mainz German Cancer Research Center (DKFZ) Heidelberg Germany; ^11^ Department of General, Visceral and Pediatric Surgery University Medical Center Göttingen Göttingen Germany; ^12^ National Center for Tumor Diseases (NCT), partner site Berlin German Cancer Research Center (DKFZ) Heidelberg Germany

**Keywords:** antigen presentation, cancer immunotherapy, immunopeptidome, mhc‐i, sumo

## Abstract

A balanced SUMOylation equilibrium safeguards the functional anti‐tumor immune response. Oncogene activation drives SUMOylation, rendering aberrant SUMOylation a hallmark of cancer. To delineate the impact of activated SUMOylation on the tumor‐immune synapse, we applied HLA class‐I‐targeted ligandomics and identified a function of activated SUMOylation in restricting the immunopeptidome landscape. Importantly, aberrant SUMOylation suppressed a unique HLA‐I peptide and oncoprotein‐derived neoepitope repertoire, enabling cancer cells to evade T cell immune surveillance. Mechanistically, SUMOylation impaired the immunoproteasome constitution and proteolytic activity, thus limiting the diversity of the peptide landscape. Further, SUMOylation altered TAP1 transporter binding preferences, thereby mimicking viral immune evasion strategies. As an actionable application, pharmacological inhibition of SUMOylation unmasked the targetable immunopeptidome, enhanced the tumor cell susceptibility to T cell killing and substantially reshaped the immune cell landscape. These findings highlight SUMOylation as a critical regulator of the adaptive anti‐tumor immune response. We propose SUMOylation inhibition as a strategy to enhance immunogenic peptide presentation, thereby improving the efficacy of cancer immunotherapies.

## Graphical abstract

Pharmacological SUMOylation inhibition (SUMOi) counteracts tumor immune evasion by unmasking an immunogenic HLA‐I peptide and neoepitope repertoire. By restoring HLA‐I ligand availability through increased antigen processing and presentation, enhanced proteasomal cleavage, and modulated TAP1 peptide affinity, SUMOi boosts tumor immunogenicity, resulting in improved T cell infiltration and cytotoxicity across multiple preclinical cancer models.

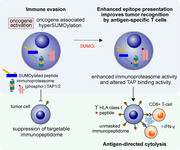

## 1 Introduction

During tumorigenesis, tumor cells rapidly adapt to evade the anti‐tumor immune response of the host immune system. T lymphocytes, especially with their cytotoxic capacity, have emerged as central players of a functional tumor‐immune synapse within the adaptive immune response. Intact antigen processing and presentation on the human leukocyte antigen HLA class‐I (HLA‐I) complex is a prerequisite for tumor recognition and killing by cytotoxic CD8^+^ T lymphocytes (CTLs) [1]. This process is a multi‐step cascade involving protein degradation by the proteasome or immunoproteasome with its catalytic subunits LMP2, LMP7 and MECL‐1, followed by transport into the endoplasmic reticulum (ER) by the transporter associated with antigen processing (TAP), a heterodimer composed of two subunits, TAP1 and TAP2 [2]. Next, cleaved peptides are loaded onto the HLA‐I complex and transported to the cell surface for antigen presentation to CTLs [1]. Notably, many tumors exhibit genetic alterations in the antigen processing and presentation machinery (APM), or transcriptionally downregulate genes encoding HLA‐I/APM pathway components, serving as main drivers of immune evasion [3, 4]. Similarly, many viruses have evolved an evolutionary conserved mechanism to escape immune surveillance by encoding viral evasion proteins that interfere with both TAP transporters, thereby blocking antigen trafficking into the ER [5]. CTLs, responsible for tumor antigen recognition and tumor cell cytolysis, are the key players in cancer immunotherapies such as immune checkpoint blockade (ICB) that depend on a functional tumor‐immune synapse. The development of ICB has transformed cancer treatment in clinical practice; however, many patients experience no or only temporary clinical benefit [6]. To date, tumor mutational burden (TMB) reflecting the global number of somatic mutations within a tumor, PD‐L1 expression, and microsatellite instability have been approved as predictive biomarkers for treatment response to ICB [7]. Remarkably, high TMB is associated with increased expression of tumor‐specific neoantigens, overall potentiating the patient's therapeutic response to ICB [8].

The balanced equilibrium of the post‐translational protein modification SUMOylation safeguards the integrity of a functional anti‐tumor immune response [9]. The conjugation of the small ubiquitin‐like modifiers (SUMOs) SUMO1, SUMO2 and SUMO3 to a target protein (SUMOylation) controls the localization, turnover and function of a target protein, thereby regulating essential cellular processes such as cell cycle progression, transcription, chromatin remodeling and DNA repair. SUMOylation is a highly dynamic process involving an enzymatic cascade of the heterodimeric E1 SUMO‐activating enzyme (SAE1/UBA2), the E2 SUMO‐conjugating enzyme (UBC9) and various E3 ligases, comprising the SUMO core machinery. SUMO1 is generally attached to its substrates as a monomer, whereas polymeric SUMO chains are formed by the highly similar SUMO2 and SUMO3 isoforms [10]. SUMOylation is fully reversible and SUMO deconjugation is catalyzed by SUMO‐specific proteases (SENPs) [11]. Activated SUMOylation is a hallmark of cancer and more generally serves as a cellular stress response [12]. Activation of oncogenes, such as *MYC*, *KRAS*, *NOTCH1* and *CTNNB1* (encoding beta‐catenin) results in upregulation of cellular SUMOylation and shifts the SUMO balance to a hyperSUMOylated state [9, 13]. We recently identified a conserved function of SUMOylation in attenuating the immunogenicity of cancer cells by suppressing antigen presentation by HLA‐I [14].

In this study, we aimed to delineate how activated SUMOylation impacts the targetable cancer immunopeptidome to confer immune evasion on several steps of antigen presentation. Our data revealed critical functions of SUMOylation in limiting peptide processing, peptide transport and, consequently, the immunopeptidome landscape of cancer cells. Our data provides a strong rationale for therapeutic targeting of SUMOylation to activate (neo)epitope processing and presentation and improve the efficacy of cancer immunotherapies.

## 2 Results

### 2.1 Oncogene‐Driven Activated SUMOylation Restricts the HLA Class‐I Immunopeptidome

**FIGURE 1:**
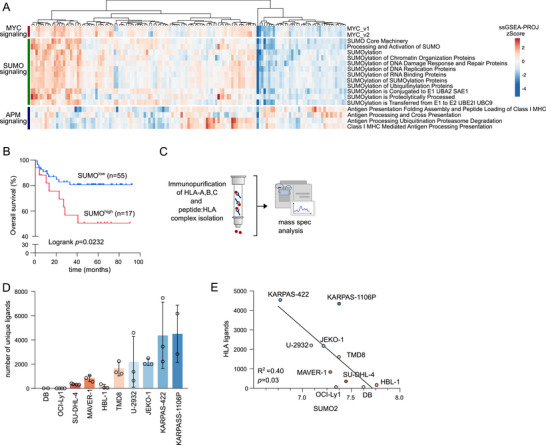
Oncogene‐driven activated SUMOylation restricts the HLA class‐I immunopeptidome. (A) Hierarchical clustering (Euclidean/Ward) of ssGSEA scores of normalized human DLBCL transcriptome profiles (GSE98588) of indicated MSigDb gene sets belonging to MYC (Hallmark), SUMO (Reactome) and the antigen presentation machinery (APM, Reactome). (B) Kaplan‐Meier‐Plot of DLBCL patient dataset (GSE34171). Patient survival has been categorized in SUMOhigh and SUMOlow based on the expression and subsequent hierarchical clustering (Euclidean/Ward) of the SUMO core machinery. Logrank p‐value is indicated. (C) Experimental strategy for the identification of HLA class‐I ligands. Peptides released upon HLA‐A/B/C immunoprecipitation were analyzed by tandem mass spectrometry. (D) Absolute count of unique HLA‐I ligands per cell in indicated B cell lymphoma cell line panel. Mean ± SD. (E) Correlation analysis of SUMO2 expression with the number of unique HLA ligands depicted in Figure 1D.

The therapeutic efficacy of ICB is often limited by an immune‐cold tumor microenvironment (TME) or loss of HLA class‐I peptide presentation, which is frequently driven by dysregulation of oncogenes [15, 16]. Previous reports linked oncogene activation to induction of SUMOylation across cancer entities [9]. Transcriptional profiling revealed a hyperSUMOylated phenotype associated with MYC signaling, suppression of the HLA‐I/APM pathway, and poor clinical outcome in human DLBCL (Figure 1A,B) and sarcoma (Figure S1A,B). To experimentally capture how activated SUMOylation fine‐tunes the tumor's immunopeptidome repertoire in lymphoma, we assessed the transcriptional profile of an informative lymphoma cell line panel and revealed substantial differences in SUMO pathway activity as indicated by differential expression of the SUMO core machinery (Figure S1C). To identify the global HLA‐I ligandome, we next performed tandem mass spectrometry (MS) of peptides eluted from immunopurified HLA‐A/B/C complexes (Figure 1C). Notably, immunopeptidomics revealed strong quantitative differences in the number of unique HLA‐bound ligands across the DLBCL cell line panel. While some cell lines exhibited high numbers of ligands, others displayed intermediate or low numbers of HLA‐I ligands, including the lymphoma cell lines DB, OCI‐Ly1, SU‐DHL‐4 (Figure 1D). High expression of the core SUMO pathway component *SUMO2* was inversely correlated with the number of unique HLA ligands per cell (Figure 1E). Further SUMO pathway components (SUMO1, SUMO3, SAE1, UBA2, UBE2I) showed similar trends but did not reach statistical significance (Figure S1D and Table S1). These findings are supported by results from a genome‐wide screening for MHC‐I regulators in DLBCL, identifying SUMO2 as a negative HLA‐I/APM regulator [17].

In summary, we identified a critical function of activated SUMOylation in suppressing the HLA class‐I immunopeptidome landscape.

### 2.2 Targeted Immunopeptidomics Reveals a Distinct Profile of SUMO‐Suppressed HLA Ligands

**FIGURE 2:**
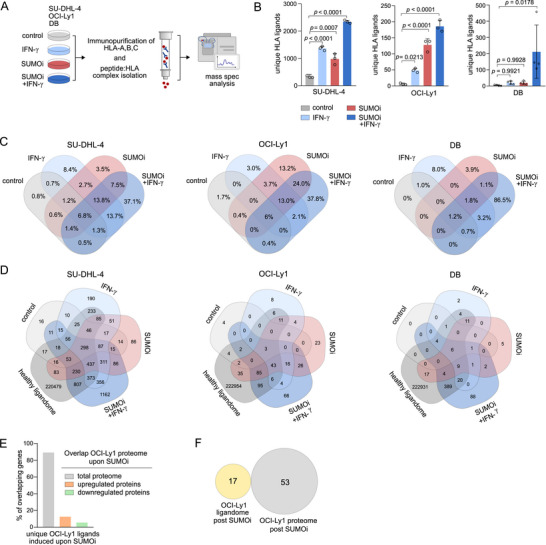
Targeted immunopeptidomics reveals a distinct profile of SUMO‐suppressed HLA ligands. (A) Experimental strategy for the identification of HLA class‐I ligands. Peptides from SU‐DHL‐4, OCI‐Ly1 and DB cells treated with control, SUMO inhibition (SU‐DHL‐4, OCI‐Ly1: SUMOi 400nM 48h, DB: SUMOi 100nM 48h), IFN‐γ (100U/ml, 24h) or the combination of both (SU‐DHL‐4, OCI‐Ly1: SUMOi 400nM 48h, DB: SUMOi 100nM 48h; IFN‐γ: 100U/ml, 24h) were released upon HLA‐A/B/C immunoprecipitation and analyzed by MS. (B) Absolute count of unique HLA‐I ligands on SU‐DHL‐4, OCI‐Ly1 and DB cells treated as depicted in (A). Mean ± SD. ANOVA with Tukey's post hoc test. (C) Venn diagrams representing the relative overlap of all peptides presented on SU‐DHL‐4, OCI‐Ly1 and DB cells in the respective treatment condition. (D) Venn diagrams representing the absolute number of all peptides on SU‐DHL‐4, OCI‐Ly1 and DB cells in the depicted treatment conditions overlapped with 223.311 HLA‐I peptides of a published dataset of healthy donors [21]. (E) Overlap of source proteins of HLA‐ligands on OCI‐Ly1 cells treated with SUMOi (400nM, 48h) with the proteome of SUMOi (400nM, 48h) treated OCI‐Ly1 cells. (F) Gene ontology (GO) enrichment analysis on source proteins of HLA‐ligands on SUMOi (400nM, 48h) treated OCI‐Ly1 cells overlapped with GO on upregulated proteins of SUMOi‐treated OCI‐Ly1 cells. Pathway analysis was performed by PantherDB functional annotation tool.

To comprehensively capture the qualitative implication of aberrant SUMOylation on the HLA‐bound ligandome, we applied the selective SUMOylation inhibitor (SUMOi) subasumstat/TAK‐981 on three DLBCL cell lines with high SUMO pathway activity (Figures S1C and S2A) and low numbers of HLA‐I ligands (Figure 1D), followed by quantitative immunopeptidomics and mass spectrometry (Figure 2A). SUMOi was administered in presence or absence of type II interferon (IFN‐γ), a cytokine known to increase the number of HLA‐I complexes on the cell surface and moreover a key player in coordinating HLA‐I antigen presentation and induction of the immunoproteasome via activation of signal transducer and activator of transcription 1 (STAT1) [18]. Consequently, tandem MS of enriched HLA‐A/B/C‐bound peptides identified 344 peptides presented on SU‐DHL‐4 control cells on average with a significant increase to 979 peptides (2.8‐fold) in SUMO‐depleted SU‐DHL‐4 cells. In OCI‐Ly1 we found a 21‐fold increase upon SUMO depletion (6 peptides to 128 peptides) and a 5‐fold increase in DB (4 peptides to 20 peptides). Similarly, IFN‐γ treatment increased the number of HLA‐I‐bound peptides by 3.9‐fold in SU‐DHL‐4, 8‐fold in OCI‐Ly1, and 5‐fold in DB cells. Furthermore, SUMOi and IFN‐γ demonstrated an at least additive effect on the induction of the immunopeptidome of all three cell lines (Figure 2B). However, intersecting the immunopeptidome of the different treatment conditions revealed distinct subsets of HLA‐I ligands highlighting that the observed differences cannot be attributed exclusively to the increase in HLA complexes (Figure 2C; Figure S2C). In line with this observation, a relevant proportion of peptides was exclusively presented in each respective treatment condition with the most unique peptides being identified upon IFN‐γ‐dependent amplification of SUMOi treatment. While 3.5%, 13.2%, and 3.9% of all peptides were exclusively presented upon SUMOi (SU‐DHL‐4, OCI‐Ly1, and DB, respectively), 37.1% (SU‐DHL‐4), 37.8% (OCI‐Ly1), and 86.5% (DB) of the immunopeptidome were unique to combined SUMOi and IFN‐γ stimulation (Figure 2C). Altogether, this may point to a highly specific suppression of HLA‐I ligands upon aberrant SUMOylation as indicated by differential presentation of HLA‐I ligands after SUMOi and IFN‐γ treatment. Similar effects were observed when investigating the impact of SUMOylation on the HLA‐I‐bound ligandome of solid cancer cell lines. Specifically, SUMOi and IFN‐γ treatment induced the immunopeptidome of the breast cancer cell line SK‐BR‐3 and the pancreatic cancer cell line PANC‐1 (Figure S2D), supporting a pivotal role of SUMOylation in restricting the targetable cancer immunopeptidome.

To explore the origin of the HLA‐I ligands, we performed pathway analyses for source proteins of SUMO‐edited versus IFN‐γ‐treated HLA class‐I ligands applying the PantherDB functional annotation [19]. As expected, HLA‐I ligands largely emanated from intracellular proteins involved in housekeeping functions including mitotic cell cycle and mRNA processing [20] (Figure S2E). Importantly, a substantial peptide fraction suppressed by activated SUMOylation originated from proteins related to immunoregulatory pathways such as the antigen presenting pathway. To further investigate the cancer specificity of the SUMO‐suppressed immunopeptidome, we matched the SUMO‐edited and IFN‐γ‐induced ligandome with over 200,000 HLA peptides from a published dataset of healthy donors that featured all HLA types of the investigated cell lines [21]. Eighty‐six peptides (<0.001%) solely presented upon SUMO depletion, and 1162 ligands (<0.01%) unique to combined SUMOi and IFN‐γ treatment in SU‐DHL‐4 have not been identified in the immunopeptidome of healthy donors (23 peptides, <0.001% upon SUMOi in OCI‐Ly1; 66 peptides, <0.001% upon combination treatment in OCI‐Ly1 and 5 peptides, <0.001% upon SUMOi in DB; 88 peptides, <0.001% upon combination treatment in DB) (Figure 2D), indicating the immunogenic potential of the unique, SUMO‐suppressed HLA ligands. As SUMOylation has an important function in protein regulation and turnover [9], we wanted to investigate if differences in the observed HLA‐I ligandome were mediated by the altered abundance of their source proteins. Thus, we overlapped the source proteins of OCI‐Ly1 ligands suppressed upon activated SUMOylation with the global proteome of SUMO‐depleted OCI‐Ly1 cells (Figure 2E). Most source proteins (89.4%) of SUMOylation‐suppressed HLA‐I peptides were detectable in the proteome analysis, whereas 12.5% of the ligands derived from upregulated and 5.6% from downregulated proteins upon inhibition of SUMOylation. Similarly, gene ontology (GO) pathway enrichment analysis on the source proteins of SUMO‐suppressed OCI‐Ly1 peptides and the SUMOi‐induced proteome revealed no overlap (Figure 2F). Taken together, these findings suggest that the differential SUMO‐regulated HLA‐I ligand presentation is not attributed to changes in protein abundance induced by SUMOi. Instead, our data highlights a distinct role of aberrant, putatively oncogenic SUMOylation in suppressing a highly unique profile of HLA peptides.

SUMOylation is a well‐established regulator of cytokine repression during immune responses with impaired SUMOylation leading to secretion of type I interferons (IFN‐I) [14, 22, 23, 24]. We next investigated the extent to which IFN‐I contributes to the immunopeptidome changes observed after SUMOi. Both IFN‐α and IFN‐β markedly expanded the immunopeptidome as well as HLA‐I surface expression (Figure S3A,B). Applying Interferon alpha/beta receptor 1 (IFNAR1) blockade (Figure S3C) to inhibit IFN‐α/β‐specific effects, the number of unique HLA‐I ligands detected after SUMOi was reduced but not completely abrogated by IFNAR1 blockade (Figure S3A). These findings indicate that although IFN‐I contributes to SUMOi‐induced antigen presentation, additional SUMO‐dependent mechanisms independently enhance the targetable immunopeptidome upon SUMOi.

### 2.3 Activated SUMOylation Restricts the HLA Peptide Landscape by Controlling Immunoproteasome Activity

**FIGURE 3:**
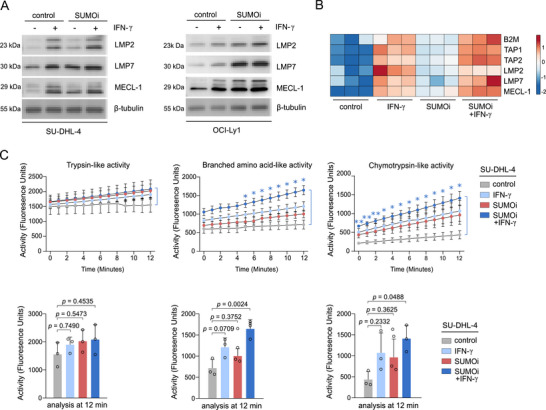
Activated SUMOylation restricts the HLA peptide landscape by controlling the immunoproteasomal activity. (A) Immunoblot analysis of SU‐DHL‐4 and OCI‐Ly1 cells treated with control, SUMOi (400nM, 48h), IFN‐γ (100U/ml, 24h) or the combination of both (SUMOi: 400nM, 48h; IFN‐γ: 100U/ml, 24h). (B) Expression of indicated proteins of the APM in OCI‐Ly1 cells, treated as depicted in (A). (C) Analysis of the immunoproteasome activity in SU‐DHL‐4 cells treated as depicted in (A) at indicated time points and displayed as bar charts at 12min. Mean ± SD. ANOVA with Tukey's post hoc test.

Immunoproteasome‐cleaved peptides serve as the major source of HLA class‐I ligands and differ from peptides released by the constitutive proteasome [25 ^–^ 27]. Given that qualitative differences in HLA‐I ligand presentation were independent of changes in protein abundance, we sought to elucidate the mechanism how activated SUMOylation modulates the HLA‐I ligand repertoire. To this end, we applied pharmacological SUMOylation inhibition as a model to systematically investigate the impact of SUMOylation on the immunoproteasome as an underlying cause of the altered HLA‐I ligand landscape. Notably, pharmacological SUMOylation inhibition enhanced the expression of the catalytically relevant subunits of the immunoproteasome LMP2, LMP7 and MECL‐1 on both transcriptional and protein level (Figure S3D,E; Figure 3A,). This effect mirrors the induction of LMP2, LMP7 and MECL‐1 observed after IFN‐γ treatment, a well‐known inducer of the immunoproteasome [28]. Importantly, SUMOi substantially amplified the IFN‐γ‐mediated induction of immunoproteasome subunits (Figure S3E). This finding was consolidated by proteome profiling of SUMOi‐ and IFN‐γ‐treated OCI‐Ly1 cells, revealing enhanced expression of the APM upon combinatory SUMOi‐ and IFN‐γ treatment (Figure 3B and Table S2). To study whether SUMO‐dependent silencing of the immunoproteasome subunits is reflected by impaired proteolytic activity of the immunoproteasome, we performed detailed analysis of the immunoproteasome's trypsin‐like, branched‐amino acid‐like and chymotryptic‐like cleavage activity in DLBCL cell lines SU‐DHL‐4 and OCI‐Ly1. Consistent with reports, IFN‐γ increased the trypsin‐like, branched‐amino acid‐like and chymotryptic‐like proteolytic activity [29] (Figure 3C; Figure S3F). Remarkably, SUMO inhibition unleashed enhanced chymotryptic‐like and branched‐amino acid‐like cleavage activity of the immunoproteasome in OCI‐Ly1 cells, that was even more pronounced upon combination with IFN‐γ in OCI‐Ly1 and SU‐DHL‐4 cells (Figure 3C; Figure S3F).

Altogether, we identified a targetable function of activated SUMOylation in controlling the activity of the immunoproteasome, thereby restricting the HLA peptide landscape.

### 2.4 SUMO Inhibition Alters Phosphorylation of TAP1, Preventing Processing and Presentation of an Immunogenic Ligandome

**FIGURE 4:**
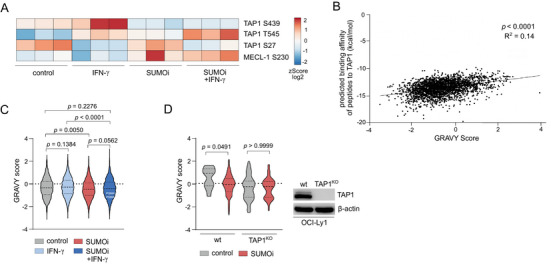
SUMO inhibition alters phosphorylation of TAP1 preventing processing and presentation of an immunogenic ligandome. (A) Heatmap depicting significant alterations in phospho‐proteomic analysis of the APM in OCI‐Ly1 cells treated with control, SUMOi (400nM, 48h), IFN‐γ (100U/ml, 24h) or the combination of both (SUMOi: 400nM, 48h; IFN‐γ: 100U/ml, 24h). (B) Correlation analysis of predicted binding affinity of HLA‐ligands on A‐375 melanoma cells [33] to TAP1. P‐value was determined by simple linear regression analysis. (C) Hydropathy analysis of HLA‐I ligands on SU‐DHL‐4 cells treated with control, SUMOi (400nM, 48h), IFN‐γ (100U/ml, 24h) or the combination of both (SUMOi: 400nM, 48h; IFN‐γ: 100U/ml, 24h), using the GRAVY score. Mean ± SD. ANOVA with Tukey's post hoc test. (D) Hydropathy analysis of HLA‐I ligands on OCI‐Ly1 wild‐type (wt) and TAP1KO cells treated with control, SUMOi (400nM, 48h), using the GRAVY score. Mean ± SD. ANOVA with Tukey's post hoc test.

To comprehensively investigate how aberrant SUMOylation restricts the functional activity of the APM pathway, we conducted phospho‐proteomic analysis of the APM in SUMOi‐treated OCI‐Ly1 cells, both in presence and absence of IFN‐γ. Common, numerous phosphorylation sites are in close proximity to SUMOylation sites and often are interconnected, highlighting the crucial role of this crosstalk in maintaining full functionality of these cellular pathways [30, 31]. Indeed, four phosphorylation sites of the APM components TAP1 and MECL‐1 were significantly altered upon changes in SUMOylation status (Figure 4A; Table S3). Specifically, SUMOylation inhibition limited phosphorylation of TAP1 (variant 2) at S439 and induced phosphorylation at T545 (Figure 4A; Table S3). Notably, these phosphorylation sites are located near critical interaction sites of highly conserved immune evasion molecules, which are encoded by viruses to inhibit TAP1 activity [32, 33], suggesting that enhanced SUMOylation dynamics in cancer may mimic viral mechanisms for TAP1‐dependent immune evasion. The TAP complex plays a conserved function in transporting peptides cleaved by the immunoproteasome from the cytosol into the lumen of the ER, where they are bound to the peptide‐loading‐HLA‐I complex [34]. Phosphorylation of TAP1 has been shown to impair its transport capacity [35]. Moreover, the TAP1's peptide binding and transport preferences strongly depend on the hydropathy of the respective peptides. We substantiated this by applying the GRAVY score (Grand Average of Hydropathy) to a published immunopeptidomics dataset to specify hydropathy of the respective peptide sequences (negative GRAVY index referring to hydrophilicity, positive GRAVY score indicating hydrophobicity). Here, we detected a significant positive correlation of the GRAVY score with calculated affinities to the TAP1 transporter [33] (Figure 4B). To investigate whether SUMO‐regulated phosphorylation of TAP affects its transporting preferences, we assessed the GRAVY score of our peptide sequences. Importantly, inhibition of SUMOylation favored presentation of peptides with a lower GRAVY index, suggesting an altered TAP binding affinity towards hydrophilic peptides in SUMO‐deprived cells (Figure 4C). To investigate the role of TAP1‐dependent peptide transport preferences in shaping the SUMO‐dependent immunopeptidome, we performed immunopeptidomics on CRISPR/Cas9‐generated TAP1‐deficient OCI‐Ly1 and control cells in presence or absence of SUMOi (Figure 4D). Importantly, we observed no changes in the GRAVY score of HLA‐I ligands following SUMOi treatment in OCI‐LY1 TAP1^KO^ cells, indicating that SUMOylation restricts HLA‐I ligand diversity at least in part through TAP1‐dependent peptide transport preferences.

In summary, these data suggest a distinct role of aberrant SUMOylation in guarding TAP1 peptide transport preferences, thereby limiting HLA‐I ligand diversity and possibly immunogenicity.

### 2.5 Aberrant SUMOylation Limits the Targetable Oncoprotein‐Derived Neoepitope Repertoire

**FIGURE 5:**
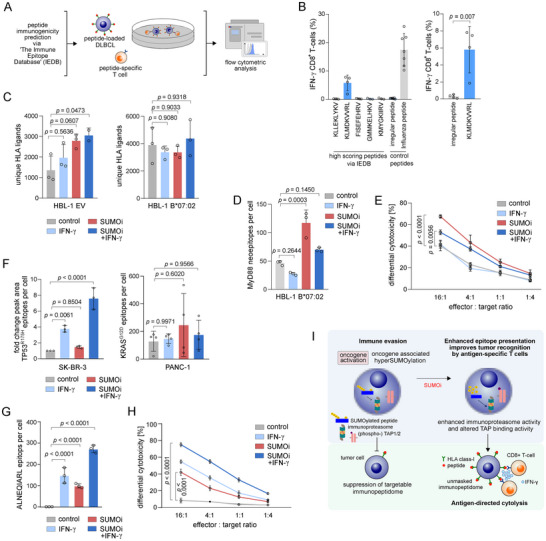
Aberrant SUMOylation limits the targetable oncoprotein‐derived neoepitope repertoire. (A) Experimental workflow to assess functional immunogenicity of SUMOi‐induced HLA‐ligands in a co‐culture model of tumor and cytotoxic T cells (CTLs). (B) IFN‐γ expression in CTLs after co‐culturing for 16hr with OCI‐Ly1 cells (loaded with 2.5M of the respective peptide for 2h) at an effector/target ratio of 5:1. Mean ± SD. Unpaired t‐test. (C) Absolute count of unique HLA ligands on HBL‐1 cells transduced with empty vector (EV) or HLA‐B*07:02 after treatment with control, SUMOi (40nM, 48h) or IFN‐γ (100U/ml, 24h) or the combination of both. Mean ± SD. ANOVA with Tukey’s post hoc test. (D) Absolute count of MyD88 L265P neoepitopes (RPIPIKYKAM) on HBL‐1 cells transduced with HLA‐B*07:02 after treatment with control, SUMOi (40nM, 48h), IFN‐γ (100U/ml, 24h) or the combination of both Mean ± SD. ANOVA with Tukey’s post hoc test. (E) Differential cytotoxicity of T cells transduced with RPIPIKYKAM‐specific TCR vs control TCR in co‐culture with HBL‐1 HLA‐B*07:02 cells treated with control, SUMOi (40nM, 48h), IFN‐γ (100U/ml, 24h) or the combination of both at indicated effector/target ratios for 16hr. Cytotoxicity was determined by LDH release assay (CytoTox 96, Promega). Pvalues at E:T 16:1 by ANOVA with Tukey’s post hoc test. (F) Changes in peak area of HMTEVVRHC neoepitope of SKBR‐ 3 cells and estimated number of VVVGADGVGK neoepitopes per cell based on spike‐in external standard heavy isotope peptides of PANC‐1 cells. Cells treated with control, SUMOi (SK‐BR‐3: 100nM, 72h; PANC‐1: 400nM, 48h), IFN‐γ (100U/ml, 24h) or the combination of both. Mean ± SD. ANOVA with Tukey’s post hoc test. (G) Absolute count of ALNEQIARL epitopes on SU‐DHL‐4 cells after treatment with control, SUMOi (400nM, 48h), IFN‐γ (100U/ml, 24h) or the combination of both Mean ± SD. ANOVA with Tukey’s post hoc test. (H) Differential cytotoxicity of T cells transduced with ALNEQIARL‐specific TCR‐mimic vs. control TCR‐mimic CAR T cells in co‐culture with SU‐DHL‐4 cells treated with control, SUMOi (400nM, 48h), IFN‐γ (100U/ml, 24h) or the combination of both. Co‐culture set‐up and readout as described in (E). (I) Schematic overview. Activated SUMOylation restricts the targetable HLA class‐I immunopeptidome, hence SUMOi unmasks the suppressed ligandome to restore tumor‐immune surveillance.

Many patients show limited response to ICB, and loss of immunogenicity has been identified as a frequent cause of resistance [18]. To systematically assess the functional immunogenicity of the unique SUMOi‐induced HLA‐I‐bound ligands, we applied a co‐culture model in which the specific interaction between tumor cells and CTLs is mediated by peptide presentation. Five HLA‐A*02 restricted ligands that were robustly induced in both OCI‐Ly1 and SU‐DHL‐4 upon SUMOi treatment and exhibited a high immunogenicity score (applying the IEDB immunogenicity prediction tool [36]) were selected for functional validation. To this end, we analyzed activation of peptide‐primed epitope specific T cells, indicated by IFN‐γ production, when co‐cultured with peptide‐loaded OCI‐Ly1 target cells (Figure 5A; Figure S4A). Indeed, KLMDKVVRL‐primed T cells produced higher levels of IFN‐γ in co‐culture compared to control peptide‐primed T cells (Figure 5B; Figure S4A), strongly indicating the immunogenic potential of SUMOi‐induced HLA‐I ligands.

Next, we explored the impact of activated SUMOylation on neoepitope presentation and immunogenicity of cancer‐specific oncogenic driver mutations. The missense mutation exchanging amino acid leucine at position 265 to proline (L265P) in the MYD88 adaptor protein is a frequent driver mutation present in about 20% of all lymphoid malignancies [37]. The neoepitope RPIPIKYKAM derived from MYD88^L265P^ has been reported to preferably bind to HLA‐B*07:02, the most common HLA‐B allele in Europe and North America [38]. We therefore transduced the MYD88‐mutant DLBCL cell line HBL‐1 to express HLA‐B*07:02 (Figure S4B). Depletion of SUMOylation increased overall peptide presentation in HBL‐1 controls, with IFN‐γ substantially amplifying the induction of the immunopeptidome, whereas the number of ligands was not significantly altered upon SUMOi in HBL‐1 HLA‐B*07:02 cells (Figure 5C). This correlated to the HLA‐I cell surface expression (Figure S4C). Notably, quantitative immunopeptidomics revealed a striking increase in MYD88^L265P^ neoepitope (RPIPIKYKAM) presentation upon SUMOi in HBL‐1 HLA‐B*07:02 cells (Figure 5D). To explore the functional consequence of enhanced neoepitope presentation upon SUMOi for T cell‐mediated tumor killing, we co‐cultured SUMOi‐treated and IFN‐γ‐treated HBL‐1 HLA‐B*07:02 cells with T cells transduced with a TCR specific for RPIPIKYKAM neoepitope recognition (TCR‐2304). Indeed, SUMOi treated HBL‐1 HLA‐B*07:02 cells demonstrated increased sensitivity towards target‐specific T cell killing compared to killing by control TCR‐transduced T cells (Figure 5E), strongly reflecting the immunogenic potential of neoepitopes unmasked by SUMO inhibition. We further expanded this analysis to SK‐BR‐3 and PANC‐1 cells, which present the TP53^R175H^‐derived, HLA‐A*02‐restricted neoepitope HMTEVVRHC (SK‐BR‐3) and the KRAS^G12D^‐derived, HLA‐A*11:01‐restricted neoepitope VVVGADGVGK (PANC‐1), respectively. Notably, SUMOi treatment led to a significant upregulation of the hydrophilic neoepitope HMTEVVRHC in SK‐BR‐3 cells, whereas no relevant increase in presentation of the hydrophobic neoepitope VVVGADGVGK was observed in PANC‐1 cells (Figure 5F). These findings support our hypothesis that altered TAP binding affinity towards hydrophilic peptides in SUMO‐deprived cells profoundly shapes the HLA‐I ligand presentation. The translational relevance of SUMOi‐driven induction of hydrophilic ligands was further demonstrated in a co‐culture model using SU‐DHL‐4 cells, which showed increased presentation of the non‐mutated, hydrophilic self‐peptide ALNEQIARL upon SUMOi (Figure 5G), an epitope targetable by TCR‐mimic CAR‐T cells. Indeed, SUMOi‐treated, ALNEQIARL‐presenting SU‐DHL‐4 cells displayed enhanced sensitivity towards target‐specific killing by TCR‐mimic CAR‐T cells (Figure 5H). In summary, we identified a distinct role of activated SUMOylation in restricting the targetable cancer immunopeptidome and specifically oncoprotein‐derived neoepitopes (Figure 5I).

### 2.6 SUMOylation Promotes Immune Evasion by Limiting the Targetable Immunopeptidome

**FIGURE 6:**
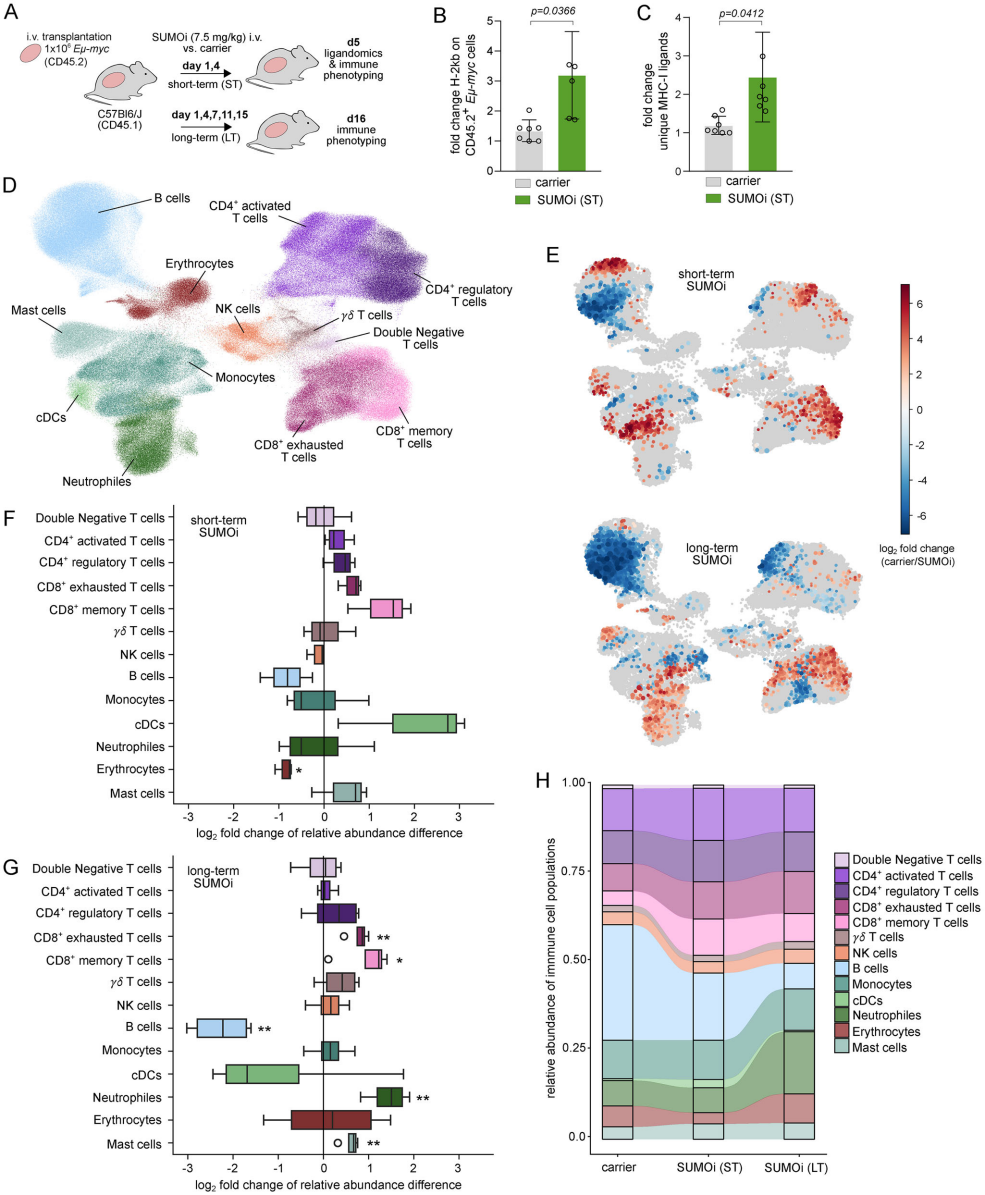
SUMOylation promotes immune evasion by limiting the targetable immunopeptidome. (A) Experimental workflow. Eμ‐myc cells (CD45.2) were transplanted into wild‐type (wt, CD45.1) recipient mice and treated with SUMOi (7.5mg/kg) or carrier on day 1, 4 (short‐term, ST) and on day 1, 4, 7, 11, 15 (long‐term, LT). (B) Normalized fold change of H‐2kb expression of Eμ‐myc cells (LN, spleen) on day 5. Mean ± SD, unpaired t‐test. (C) Normalized fold change of MHC‐I ligands of Eμ‐myc cells (LN, spleen) on day 5. Mean ± SD, unpaired t‐test. (D) UMAP visualization of 36‐plex cytometry data of LNs from experiment shown in (A). (E) Detection of differentially abundant cell populations in LN of carrier, ST and LT SUMOi‐treated mice using Milo‐DA package [76]. Red: greater abundance after SUMOi, blue: greater abundance after carrier. (F, G) Boxplots indicating differential abundance of LN cell populations, comparing abundances after ST (F), LT (G) SUMOi with carrier treatment. Positive log2 FC: population is more abundant after SUMOi. (H) Alluvial plot depicting changes of immune cell type abundance over time.

Finally, to test the in vivo potential of SUMOi as a strong inducer of the MHC‐I peptide landscape, we transplanted primary *Eμ‐myc* lymphoma cells [40] into syngeneic wildtype recipient mice, treated with SUMOi or carrier solution, and performed immunopeptidomics (Figure 6A). In support of our previous findings, SUMOi significantly enhancing MHC‐I (H‐2 kb) expression on the CD45.2^+^ *Eμ‐myc* lymphoma cells (Figure 6B). Importantly, immunopeptidome analysis revealed a significant upregulation of MHC class‐I peptides on *Eμ‐myc* lymphoma cells after in vivo SUMOi treatment (Figure 6C).

To investigate how the SUMO‐limited targetable immunopeptidome impacts the immune cell landscape, we performed 36‐plex spectral flow cytometry on the lymph nodes’ immune cell landscape from SUMOi‐treated E*μ*‐*myc* lymphoma mice on two different time points (Figure 6A). This analysis provided a high‐resolution cellular landscape, profiling all major immune cell types and activation states and capturing a total of 443.540 high‐quality cells (Figure 6D). Importantly, we observed increases in CD4^+^ and CD8^+^ T cells after short‐term SUMO inhibition (Figure 6E–H). These trends became more prominent after long‐term SUMOi exposure showing a significant increase in CD8^+^ memory T cells. Besides, healthy B cells were less abundant, whereas neutrophils and mast cells were markedly increased in SUMOi‐treated mice. In line with the prominent immuno‐modulatory changes following SUMO inhibition and previous findings [41], we observed a decline in tumor burden (Figure S4D). This data was supported by CIBERSORTx analysis [42] of RNAseq data from a representative aggressive B cell lymphoma cohort  [43]. By stratifying samples into a SUMO^high^ and SUMO^low^ cohort, we observed a significant higher proportion of tumor‐infiltrating CD8^+^ T cells in the SUMO^low^ patient subgroup (Figure S4E), supporting the role for SUMOylation as a critical regulator of the antitumor immune response.

In summary, we identified a distinct role of activated SUMOylation in restricting the targetable cancer immunopeptidome, thereby impairing the antitumor immune response. Altogether, we propose pharmacological SUMO inhibition as a novel strategy to enhance immunogenic epitope presentation to advance therapeutic efficacy of cancer immunotherapies or TCR‐based cellular therapies.

## 3 Discussion

Immune surveillance provides an effective barrier to tumorigenesis and tumor progression that is safeguarded by a balanced equilibrium of SUMOylation [44]. Oncogene activation drives SUMOylation, compromising the integrity of a functional anti‐tumor immune response [9]. In this study, we unraveled a critical function of activated SUMOylation in limiting the cancer immunopeptidome, thereby disrupting the integrity of a functional tumor‐immune interface. Consequently, we propose SUMO inhibition as a potential strategy to enhance the efficacy of cancer immunotherapies by unmasking the targetable immunopeptidome.

Recent reports linked the presence and diversity of a functional immunopeptidome to clinical responses to cancer immunotherapies like ICB [45, 46]. To date, high TMB reflecting the global number of somatic mutations within a tumor serves as a surrogate parameter for enhanced expression of tumor specific antigens, thereby predicting the patients’ therapeutic response to ICB [7]. By now, high TMB has been approved as a tumor‐agnostic biomarker for ICB pembrolizumab in second‐line treatment for metastatic solid tumors. Given the clinical challenge that only subgroups of patients respond to ICB, a deeper understanding which mechanism constrains the immunopeptidome landscape is of utmost importance. Hence, strategies aimed at increasing the diversity of peptide presentation, therefore mimicking or inducing a TMB‐high‐like state, hold significant promise for enhancing efficacy of ICBs.

By utilizing HLA class‐I immunopeptidomics, we identified not only a unique repertoire of SUMO‐suppressed HLA‐I peptides, but revealed a novel function of aberrant SUMOylation in suppression of oncogene‐derived neoepitopes. Mechanistically, SUMOylation impaired the proteolytic activity of the immunoproteasome, thereby limiting the diversity of the peptide landscape. This is consistent with reports that immunoproteasome‐cleaved peptides serve as the major source of HLA class‐I ligands and differ from peptides released by the constitutive proteasome [25, 27]. To delineate how activated SUMOylation restricted the functional activity of the immunoproteasome and APM pathway, we conducted phospho‐proteomic analyses. SUMOylation induced phosphorylation of TAP1 at a position essential for peptide binding and transport. This phosphorylation site is located near key interaction regions targeted by highly conserved viral immune evasion molecules designated to block TAP1 activity [32, 33]. Thus, aberrant SUMOylation may mimic viral evasion patterns, ultimately leading to diminished presentation of qualitatively altered peptides, similar to the effects observed with TAP1 mutations in cancer [47]. In support of this, we identified the hydrophobicity of HLA ligands as a surrogate parameter for the affinity of peptides to TAP1, which significantly changes upon modifications in SUMOylation status, an effect that was abrogated in TAP1‐deficient cells. This finding supports recent evidence highlighting the critical role of the peptide loading complex in regulating the presented HLA‐I ligand repertoire [48]. However, it is important to note that further analyses are needed to fully dissect the precise molecular mechanisms underlying the SUMOylation dependent effects on TAP‐1 binding preference. Endogenous retroviral elements (ERVs) might be considered as an alternative source of targetable HLA‐I ligands, as these genomic regions have been reported to be presented as HLA‐I ligands [49] and can be activated upon SUMO inhibition [50]. However, a comprehensive assessment of ERV‐derived ligands by 5’RACE sequencing is a topic of future studies to determine which ERV transcripts are specifically induced upon SUMO inhibition.

Translating our findings to potential clinical application, we demonstrated that pharmacological inhibition of SUMOylation unmasked the clinically relevant immunopeptidome and increased the tumor cell susceptibility to CD8^+^ T cell killing. Thus, we suggest SUMOi as a valid therapeutic strategy to enhance the efficacy of cancer immunotherapies that rely on a functional tumor‐immune synapse like ICB or TCR T cell therapy. Previously, inhibition of EZH2, CDK4/6 and ALK were reported to modulate the immunopeptidome [39, 51, 52], and moreover to enhance immunotherapeutic activity in a preclinical mouse model [53, 54]. Our findings thus strengthen this growing body of research on posttranslational and epigenetic mechanisms that can be pharmacological targeted to restore tumor cell immunogenicity [18, 55], and propose a novel strategy.

Targeting SUMOylation is of particular interest as activated SUMOylation has been identified as a characteristic of many cancer entities and especially following oncogene activation [9, 13, 56]. Importantly, pharmacological inhibition of SUMOylation significantly enhanced the presentation of hydrophilic public neoepitopes derived from MyD88^L265P^and TP53^R175H^ mutations and a non‐mutated NDC80‐derived target peptide whereas a strongly hydrophobic peptide from KRAS^G12D^ was not significantly induced. For MyD88^L265P^ and the NDC80‐derived targets T cell‐mediated cytolysis was increased accordingly in the SUMOi‐treated samples. This finding could be of great clinical importance, as the MyD88 adaptor protein missense mutation is one of the most common driver mutations, present in about 20% of all lymphoid malignancies [37]. Upregulation of neoepitopes by pharmacological drug treatment has been observed previously, while data clearly outline how pharmacological targeting improves sensitivity towards neoepitope‐specific TCR T cells [57].

Importantly, we identified a distinct role of activated SUMOylation in restricting the targetable cancer immunopeptidome, thereby impairing the antitumor immune response. These findings highlight pharmacological SUMO inhibition as a promising strategy to enhance immunogenic epitope presentation and improve therapeutic efficacy of cancer immunotherapies and T cell based cellular therapies. Our results align with a growing body of evidence demonstrating profound remodeling of the immune system following SUMOi [14, 58 ^–^ 60]. Given the pronounced effects of SUMO inhibition on T cell infiltration and activation, its combination with immune checkpoint blockade has showed strong synergistic activity in two independent mouse models [24]. Moreover, SUMO inhibition has entered clinical evaluation and demonstrated no dose‐limiting toxicities [61].

In this study we unraveled the SUMO pathway as a coordinator of HLA class‐I ligand presentation and peptide diversity in cancer. Activity of the SUMO pathway might serve as a predictive biomarker for the diversity of the HLA‐I immunopeptidome, aiming to estimate the responses to ICB. Moreover, we here provided mechanistic insight how the HLA‐I ligandome is regulated and how it parallels viral immune evasion strategies. From a clinical perspective, highly immunogenic tumor cells are often targeted and eliminated by the immune system in a process known as immunoediting, which promotes the survival of less immunogenic cancer cells and contributes to resistance against cancer immunotherapies [62]. Enhancing the immunogenicity of tumor cells is an established key therapeutic goal, and we here propose SUMO inhibition as a promising strategy to boost or reestablish the effectiveness of cancer immunotherapies.

## 4 Conclusion

Despite the broad success of cancer immunotherapies in clinical practice, therapeutic efficacy is often limited by loss of tumor immunogenicity, underscoring the importance of a deeper understanding of tumor immune evasion mechanisms. Here, we report a function of activated oncogenic SUMOylation in shielding tumors against immune destruction by restricting the targetable immunopeptidome landscape. We provide mechanistic insight into how aberrant SUMOylation impairs function and activity of the antigen processing and presentation machinery. Given the therapeutic potential of enhancing tumor immunogenicity, we identify pharmacological inhibition of SUMOylation as a rational therapeutic strategy for unmasking a unique HLA‐I peptide and neoepitope repertoire to boost the efficacy of cancer immunotherapies.

## 5 Material and Methods

### 5.1 Chemicals

TAK‐981 was either purchased from Med Chem Express or provided by Millennium Pharmaceuticals, Inc., a wholly owned subsidiary of Takeda Pharmaceutical Company Limited. TAK‐981 doses and treatment durations are indicated in the figure legends. Recombinant human IFN‐γ was purchased from PeproTech. For IFN‐γ‐induced HLA class‐I induction, cells were treated with 100U/ml for 24 h.

### 5.2 Cell Culture

Human DLBCL cell lines were kept in RPMI‐1640: SU‐DHL‐4 (RRID:CVCL_0539), HBL‐1 (RRID:CVCL_4213), MAVER‐1 (RRID:CVCL_1831), TMD8 (RRID:CVCL_A442), U‐2932 (RRID:CVCL_1896), JEKO‐1 (RRID:CVCL_1865), KARPAS‐422 (c), KARPAS‐1106P (RRID:CVCL_1821), DB (RRID:CVCL_1168) or DMEM: PANC‐1 (RRID:CVCL_VQ69) or McCoy's 5a: SK‐BR‐3 (RRID:CVCL0033) or IMDM: OCI‐Ly1 (RRID:CVCL_1879) medium supplemented with 10% FCS, and 2 mM L‐glutamine. All cell lines were authenticated and confirmed to be contamination‐free.

### 5.3 Viral Transfection and Transduction

Lentiviral HLA‐B*07 pLEX_307 plasmids (kindly provided by B. Chapuy, Department of Hematology, Oncology and Cancer Immunology, CBF, Berlin, Germany) were cotransfected with lentiviral helper plasmids for virus production into HEK‐293 cells (Lipofectamin, Thermo Fisher Scientific). Virus supernatants were used to spinfect HBL‐1 cells in the presence of 8 µg/mL polybrene (Sigma‐Aldrich). At 96 h post‐infection, cells were selected for puromycin resistance.

### 5.4 Generation of NDC80‐CAR T Cells and LDH Killing Assay

The CAR sequence was cloned into a pCDH lentiviral vector (Systems Biosciences) for delivery into T cells. Human T cells were activated with CD3/CD28 Dynabeads (Thermo Fisher Scientific, 11‐161‐D). One day after activation, human T cells were transduced with concentrated lentivirus in RetroNectin (Takara) coated plates. Transduced T cells were then expanded in the presence of 100 U/mL IL2 (Sigma) for 8–12 days. Transduction efficiency was assessed by direct staining using anti‐myc clone 71D10‐Al647 (Cell Signaling #63730). For killing assays 10,000 target cells were incubated for 16–18 h with NDC80 TCRmimic CAR T cells. The assay was used according to the manufacturer's protocol.

### 5.5 Flow Cytometry

Cells were washed in HF2 buffer (ddH2O, 2% FCS, 1% P/S, 1% HEPES, 10% HBSS) and stained on ice for 30 min in HF2 (a list of all antibodies is provided in the supplements). After washing in HF2, cells were either resuspended in DPBS containing DAPI for FACS analysis or fixed with BD Biosciences Cytofix/Cytoperm for intracellular staining. Data were acquired on Beckman Coulter CytoFLEX S.

### 5.6 Immunoblot Analysis

Protein extracts were prepared by solving cell pellets in lysis buffer (150 mM NaCl, 1% NP‐40, 0.5% sodium deoxycholate, 0.1% SDS, 50 mM Tris) supplemented with NaF, PMSF, and NaVO4. Protein lysates were fractioned on SDS PAGE gels, transferred to PVDF transfer membrane (Thermo Fisher Scientific), and incubated with specific antibodies. A list of all antibodies is provided in the supplements.

### 5.7 Immunoproteasome Activity Assay

The proteolytic activity of the (immuno) proteasome was evaluated using the immunoproteasome activity fluorometric assay kit II (UBPBio, Dallas, Tx, USA), as described by the manufacturer. In brief, 300 µg protein extract was incubated in the provided buffer with fluorophore‐linked peptide substrate (LLVY‐7‐amino‐4‐methylcoumarin [AMC], 100 µM) at 37°C. Proteasome activity was measured over time by quantification of relative fluorescent units from the release of the fluorescent‐cleaved product AMC using a 360/40 nm filter set in a fluorometer (Synergy LX Reader, Biotek). A solution of the lysate of murine splenocytes or DLBCL cells and the proteasome inhibitor MG‐132 (100 µM) were used as a negative control.

### 5.8 CRISPR/Cas9‐Based Gene Editing

To deplete TAP1 in OCI‐Ly1, Exon 1 was removed by CRISPR/Cas9 gene editing. 150,000 cells were transfected with 500 ng of each of the sgRNA's and 1 µg Cas9 protein (PNA Bio) with a Neon Transfection System (Thermo Fisher/Invitrogen) (parameters: 1450 V; 10 ms; 3 pulses) (sgRNA_TAP1_1: GGCCTAGAAGCCGACGCACA, sgRNA_TAP1_2: GGACGGCGTCCGAGTGCCAA). The cleavage efficacy was tested 24 h after transfection with the Terra PCR Direct mix and primers flanking the target exon. Cells were then separated into single cells by serial dilution. Cell clones were screened for efficient gene editing and selected for TAP1 expression.

### 5.9 RNA‐seq and Processing of Gene Expression Data

 RNA isolation was conducted as described for reverse transcription qPCR. RNA concentration and purity were assessed using the NanoDrop equipment (NanoDrop Technologies Inc., Wilmington, DE). Library preparation and paired‐end sequencing were performed by Novogene on a HiSeq2500 (Illumina) with a sequencing depth of more than 20 M reads/sample. The resulting Fastq files were mapped to the human reference genome hg38 and counted using STAR. Counts were normalized and differential gene expression was analyzed by DESeq2. Normalized count tables were subsequently used for gene set enrichment analysis (GSEA), using the Kolmogorov‐Smirnov test and gene ontology biological process signatures of the Molecular Signature Database [63], implemented in GeneTrail 3.2 [64]. Selected gene expression results were illustrated in heatmaps using ClustVis [65]. For Supplementary Figure S3D, FastQ files were trimmed using fastp (v. 0.22.0) [66] and mapped to the human reference genome hg39 using salmon (v. 1.10.3) [67]. Count matrices were generated with tximport (v. 1.34.0) [68] using the parameter countsFromAbundance = “lengthScaledTPM” followed by normalization with DESeq2 (v. 1.46) [69]. Normalized gene expression values were z‐transformed and visualized as a heatmap.

### 5.10 Deep phosphoproteome Profiling Using TMT‐Based Mass Spectrometry

Cell pellets were resuspended in lysis buffer (1% Sodium deoxcholate, 100 mM Tris‐HCl pH 8, 150 mM NaCl, 1 mM EDTA, 40 mM CAA, 10 mM DTT, phosphatase inhibitor cocktail 2 and 3 (Sigma)), heated for 10 min at 95°C, cooled down and treated with Benzonase (Merck, 50 units) for 30 min at 37°C. Endopeptidase LysC (Wako) and sequence‐grade trypsin (Promega) were added to 100 µg protein extract (enzyme‐to‐protein ratio of 1:50), followed by an incubation over night at 37°C. Peptides were desalted, resuspended in 50 mM HEPES and labeled with 16‐plex tandem mass tag reagents (TMTpro, Fisher Scientific) following the vendors instructions. Samples were combined, desalted, fractionated by high‐pH reversed phase off‐line chromatography (1290 Infinity, Agilent) and pooled into 30 fractions. 10% of each fraction was used directly for global proteome measurements. The remaining 90% were further pooled onto 15 fractions and applied to IMAC based phosphopeptide enrichment using Fe(III)‐IMAC cartridges and the AssayMAP Bravo Platform (Agilent Technologies).

For LC‐MS/MS measurements, peptides were reconstituted in 3% acetonitrile with 0.1% formic acid and separated on a reversed‐phase column (20 cm fritless silica microcolumns (inner diameter of 75 µm, packed with ReproSil‐Pur C18‐AQ 1.9 µm resin (Dr. Maisch GmbH)), using a 98‐min gradient of increasing Buffer B (90% ACN, 0.1% FA) concentration (from 2% to 60%) with a 250 nL/min flow rate on a High‐Performance Liquid Chromatography (HPLC) system (Thermo Fisher Scientific) and analyzed on an Q Exactive HF‐X instrument (Thermo Fisher Scientific). The mass spectrometer was operated in data‐dependent acquisition mode using 60K resolution, 350–1500 m/z scan range, maximum injection time of 10 ms. The top 20 MS/MS scan were obtained at 45K resolution with 0.7 m/z isolation window and a maximum injection time of 86 ms. Dynamic exclusion was set to 30 s and only precursor with a charge state between 2–6 were selected for fragmentation. For phosphopeptide enriched samples the maximum injection time was increased to 120 ms.

RAW data were analyzed with MaxQuant software package (v 1.6.10.43) using the Uniprot human databases (UP000005640_2022_03). The search included variable modifications of methionine oxidation and N‐terminal acetylation, deamidation (N and Q) phosphorylation (STY) and fixed modification of carbamidomethyl cysteine. Reporter ion MS2 for TMT16 was selected (internal and N‐terminal) and TMT batch specific corrections factors were specified. The FDR (false discovery rate) was set to 1% for peptide and protein identifications. Data were filtered for 100% valid value. Reverse hits, contaminants and hits only identified by site were excluded. Reporter intensities were log2 transformed and z‐score normalized. Differential abundance was calculated using Student's t‐test and a significance cut‐off of FDR5% (or 1%).

### 5.11 Immunopurification of HLA Class‐I Ligands

For immunopurification suspension cells were harvested through direct resuspension. Harvested cells were pelleted and washed three times in ice‐cold sterile PBS (21‐040‐CM). For each replicate 50*10^6^ cells were used. Cells were lysed in 7.5 mL of 1% CHAPS (Sigma‐Aldrich, Cat# C3023) dissolved in PBS and supplemented with protease inhibitors (cOmplete, Cat# 11836145001). Cell lysis was performed for 1 h at 4°C, lysates were spun down for 1 h with 20,000 g at 4°C, and supernatant fluids were isolated. Affinity columns were prepared as follows: 40 mg of Cyanogen bromide‐activated‐Sepharose 4B (Sigma‐Aldrich, Cat# C9142) were activated with 1 mM hydrochloric acid (Sigma‐Aldrich, Cat# 320331) for 30 min. Subsequently, 1 mg of W6/32 antibody (BioXCell, Cat #BE0079) was coupled to sepharose in the presence of binding buffer (150 mM sodium chloride, 50 mM sodium bicarbonate, pH 8.3; sodium chloride: Sigma‐Aldrich, Cat# S9888, sodium bicarbonate: Sigma‐Aldrich, Cat#S6014) for at least 2 h at room temperature. Sepharose was blocked for 1 h with glycine (Sigma‐Aldrich, Cat# 410225) and washed 3 times with PBS. Supernatants of cell lysates were run over the columns through peristaltic pumps with a 1 mL/min flow rate overnight in a cold room. Affinity columns were washed with PBS for 30 min, water for 30 min, then run dry, and MHC complexes were subsequently eluted five times with 200 µl 1% trifluoracetic acid (TFA, Sigma/Aldrich, Cat# 02031). The TFA eluates were pooled and loaded onto C18 columns (Sep‐Pak C18 1 cc Vac Cartridge, 50 mg Sorbent per Cartridge, 37–55 µm Particle Size, Waters, Cat# WAT054955) which were prewashed with 80% acetonitrile (ACN, Sigma‐Aldrich, Cat# 34998) in 0.1% TFA and equilibrated with two washes of 0.1% TFA. Samples were loaded, washed again with 0.1% TFA and eluted in 400 µl of each 30%, 40%, and 50% ACN in 0.1%TFA. The sample volume was reduced by vacuum centrifugation for mass spectrometry analysis. For quantitative mass spectrometry custom synthesized AQUA heavy peptides with >90% purity (Thermo Fisher) were spiked in at 30 fmol. Peptide sequences included VVVGADGVGK (+8 Da) and ALNEQIAR(+10)L.

### 5.12 Solid Phase Extractions (SPE)

In‐house C18 mini columns were prepared as follows: for SPE of one sample two small disks of C18 material (1 mm in diameter) were punched out from CDS Empore C18 disks (Fisher Scientific, Cat# 13‐110‐018) and transferred to the bottom of a 200 µl Axygen pipette tip (Fisher Scientific, Cat# 12639535). Columns were washed once with 100 µl 80%ACN/0.1%TFA and equilibrated with 3 times 100 µl 1%TFA. All fluids were run through the column by centrifugation in mini tabletop centrifuges and eluates were collected in Eppendorf tubes. Then, dried samples were resuspended in 100 µl 1%TFA and loaded onto the columns, washed twice with 100 µl 1%TFA, ran dry, and eluted with 50 µl 80%ACN/0.1% TFA. Again, the sample volume was reduced by vacuum centrifugation.

### 5.13 LC‐MS/MS Analysis of MHC Ligands

Samples were analyzed by LC‐MS/MS (Orbitrap Exploris, Thermo Fisher). 480 mass spectrometers (Thermo Fisher Scientific). A 44 min gradient was applied using an EASY‐nLC 1200 system (Thermo Fisher Scientific) with an in‐house packed column (C18‐AQ 1.9 µm beads; Dr. Maisch Reprosil‐Pur 120). MS1 resolution was set to 120’000, a MIPS peptide filter with relaxed restrictions was applied, the minimum intensity threshold was specified to 50’000, dynamic exclusion occurred for 20 s and charge states 1–5+ were allowed as precursors. For MS/MS, the maximum injection time was 100 ms with an AGC of 50,000 in a data‐dependent acquisition mode.

### 5.14 Mass Spectrometry Data Processing and HLA Assignments

Mass spectrometry data were processed using Byonic software (version 4.5.2, Protein Metrics, Palo Alto, CA) through a custom‐built computer server. Mass accuracy for MS1 was set to 10 ppm and 20 ppm for MS2, respectively. Digestion specificity was defined as unspecific and only precursors with charges 1,2, 3, and up to 2 kDa were allowed. Protein FDR was disabled to allow a complete assessment of potential peptide identifications. Oxidation of methionine, phosphorylation of serine, threonine, and tyrosine as well as cysteinylation of cysteine were set as variable modifications for all samples. Samples were searched against the UniProt Human Reviewed Database with common contaminants added. Peptides were selected with a minimal log prob value of 1.3 indicating p‐values for PSMs of <0.05 and duplicates were removed. Then netMHCpan 4.1 algorithm with default settings was applied considering peptides with %rank <0.5 strong and %rank <2 weak binders. For quantitative analyses Skyline software (Version 25.1) was used.

### 5.15 Generation of Peptide‐Specific CD8^+^ T Cells

Production of peptide‐specific CD8^+^ T cells was adapted from Woelfl et al. [70]. Heparinized peripheral blood samples were obtained from HLA‐A2–positive healthy individuals. PBMCs were isolated by Ficoll density gradient centrifugation. CD8 separation was done by MACS MicroBeads isolation. The CD8^−^ fraction was kept in 6‐well plates for 2 h to allow adherence to plastic. Adherent cells were cultured in presence of GM‐CSF (800 U/mL) and IL‐4 (1000 U/mL) for 48 h to obtain DCs. DC maturation was induced by adding IL‐4 (1000 U/mL), LPS (10 ng/mL), and IFN‐γ (100 U/mL) overnight. Mature DCs were then loaded with the specific peptide (2.5 µg/mL) for 2 h at 37°C before coculturing with CD8^+^ cells (ratio DC/CD8^+^  = 1:10) in the presence of IL‐7 and IL‐15 (5 ng/mL) for 10 days. Medium and cytokines were replaced every 2 to 3 days.

### 5.16 T Cell Activity Assay

Cancer cells were loaded with the respective peptides (2.5 µg/mL) for 2 h at 37°C. After washing, cells were co‐cultured with peptide‐specific CTLs or control CTLs at indicated effector/target ratios. IFN‐γ production of CD8^+^ T cells was analyzed using flow cytometry.

### 5.17 Animal Experiment

Wildtype (wt) mice (CD45.1, C57Bl/6J) were obtained from the Jackson Laboratory. 1 × 10^6^ *Eμ‐myc* lymphoma cells (CD45.2) were transplanted intravenously (i.v.) into C57Bl/6J wt mice. Upon tumor development, mice were randomized and i.v. treated with 7.5 mg/kg SUMOi or carrier control on day 1, 4 (short‐term) or on day 1, 4, 7, 11, 15 (long‐term). Mice were euthanized 24 h after receiving the second dose. All animal experiments were performed in accordance with local authorities (LAGeSo Berlin).

### 5.18 Flow Cytometric Data Preprocessing

Similar as reported before [71], the full‐spectrum flow cytometry data raw FCS files were spectrally unmixed using the inbuilt unmixing function of the SpectroFlo (Cytek Biosciences) software. FCS files were imported into FlowJo (BD) to assess unmixing by visualizing N × N plots. Axes were adjusted wherever needed and parameters for logicle [72] or generalized bi‐exponential transformation of data were defined for every surface marker individually. FlowAI [73] was used as an automatic quality control mechanism for cytometry data where needed. The populations of interest were exported using channel values defined by the inbuilt export function of FlowJo.

### 5.19 Flow Cytometric Data Analysis

Full spectrum flow cytometry data was further analyzed with the scverse [74] ecosystem for dimensional reduction tasks such as PCA and UMAP generation with the scanpy and anndata packages [75]. Differential abundance analysis was performed with the pertpy implementation of milo [76]. Alluvial plots were generated with ggalluvial [77].

### 5.20 CIBERSORTx Analysis

Affymetrix expression data were downloaded from GEO (GSE4475). Cell type abundances were estimated using CIBERSORTx [42] (with the LM22 signature matrix. Gene set variation analysis was performed using the GSVA package (v2.0.7) [78] with the CORE_SUMO_SIGNATURE defined as: UBE2I, SAE1, UBA2, SUMO1, SUMO2, and SUMO3. Data were visualized using a boxplot, and group means were compared using the Wilcoxon test.

### 5.21 Statistical Analysis

Statistical analyses were performed using GraphPad Prism (GraphPad Software, La Jolla, CA). The error bars shown in the figures represent the standard deviation (SD), unless specified otherwise. A *p*‐value lower than 0.05 was generally considered significant and all exact P‐values and tests are indicated in the figures.

## Author Contributions

Conception and design of the study by U.M.D, A.M., S.M., M.W., M.G.K. and U.K. Acquisition of data and/or analysis and interpretation of data by U.M.D., A.M., B.Z., B.A., D.S., L.V.H., K.I., C.Z., M.K., S.M., S.Y., S.M., M.K., S.H., A.B., P.M., S.M., M.W., M.G.K. and U.K. Drafting of the manuscript by U.M.D., A.M., M.G.K. and U.K. All authors revised the manuscript for important intellectual content and approved the final version submitted for publication.

## Funding

This work was supported by Deutsche Forschungsgemeinschaft (DFG) grants KE222/11‐1 to U.K., KE222/10‐1 to U.K. and S.M., MU1764/7‐1 to S.M., WI 6148/1‐1 to M.W.; Deutsche Krebshilfe grants 70114425 to U.K., 70114724 to U.K. and S.M., 70114823 to S.M., 70115444 to U.K. and M.W.; Hector Foundation grant M2408 to M.W. and M.G.K., grant M2506 to U.K.; Wilhelm Sander Stiftung grant 2024.109.1 to U.M.D.; Else Kröner‐Fresenius‐Stiftung grant 2024_KEA.07 to U.M.D.; DKTK partner site Berlin young investigator grant to M.G.K. and Stiftung Charité to U.K. U.M.D. and M.G.K. are participants in the BIH‐Charité Clinician Scientist program funded by the Charité‐Universitätsmedizin Berlin und BIH.

## Conflicts of Interest

U.K. received reimbursement for advisory board function, speaker honorary and travel support from Takeda for content unrelated to this manuscript. M.G.K. is a scientific advisor to Ardigen, T‐knife and BioCopy with no conflicts of interest related to this manuscript. All other authors report no conflicts of interest.

## Supporting information




**Supporting File**: advs73832‐sup‐0001‐SuppMat.pdf.

## Data Availability

The immunopeptidome data generated in this study have been deposited on the PRIDE server with the data set identifier PXD061196. The MS proteomics data have been stored on the PRIDE server with the data set identifier PXD060641. The RNA Seq data used in this study have been stored on the European Nucleotide Archive (ENA) with the accession number PRJEB49824 and PRJEB53800.
