# Supplementary material for: Aberrant SUMOylation Restricts the Targetable Cancer Immunopeptidome
Source: Adv Sci (Weinh). 2026 Jan 21;13(17):e11449. doi: 10.1002/advs.202511449 (PMC13042404; doi:10.1002/advs.202511449)
Supplement: Supplementary file 1 — Supporting File: advs73832‐sup‐0001‐SuppMat.pdf. [file ADVS-13-e11449-s001.pdf]

SUPPLEMENTARY FIGURES

Aberrant SUMOylation restricts the targetable cancer immunopeptidome

Uta M. Demel[1,2,3], Anna Meurer[1,2], Badeel Kh. Q. Zaghla[1], Bilge Atay [1], Daniel Steiert[1,2,4], Luca V. Hummel[1,2], Konstandina Isaakidis[1,2], Chuanbing Zang[1,2], Michael Korenkov[1,2], Marlon Schielin[1,2], Schayan Yousefian[1,4,5], Shima Mecklenbräuker[1], Marieluise Kirchner[2,5], Simon Haas[1,4,5,6,7,8], Antonia Busse[1,2,6], Philipp Mertins[2,5,6], Stefan Müller[9,10], Matthias Wirth[1,2,6,11], Martin G. Klatt[1,3,6‡] and Ulrich Keller[1,2,6,7,12‡]

[1]Department of Hematology, Oncology and Cancer Immunology, Charité - Universitätsmedizin Berlin, corporate member of Freie Universität Berlin and Humboldt-Universität zu Berlin, 12203 Berlin, Germany.
[2]Max-Delbrück-Center for Molecular Medicine, 13125 Berlin, Germany.
[3]Clinician Scientist Program, Berlin Institute of Health (BIH), Berlin, Germany.
[4]Berlin Institute for Medical Systems Biology, Max Delbrück Center for Molecular Medicine in the Helmholtz Association, Berlin, Germany.
[5]Berlin Institute of Health (BIH) at Charité – Universitätsmedizin Berlin, 10117 Berlin, Germany.
[6]German Cancer Consortium (DKTK) partner site Berlin, German Cancer Research Center (DKFZ), 69120 Heidelberg, Germany.
[7]Cluster of Excellence ImmunoPreCept, Charité - Universitätsmedizin Berlin, Berlin, Germany.
[8]Precision Healthcare University Research Institute, Queen Mary University of London, London, UK.
[9]Institute of Biochemistry II, Goethe University Frankfurt, Medical School, 60590 Frankfurt, Germany.
[10]German Cancer Consortium (DKTK) partner site Frankfurt/ Mainz, German Cancer Research Center (DKFZ), 69120 Heidelberg, Germany.
[11]Department of General, Visceral and Pediatric Surgery, University Medical Center Göttingen, 37075 Göttingen, Germany.
[12] National Center for Tumor Diseases (NCT), partner site Berlin, German Cancer Research Center (DKFZ), 69120 Heidelberg, Germany

[‡]Equal contribution

*Corresponding Author: Ulrich Keller, Department of Hematology, Oncology and Cancer Immunology, Campus Benjamin Franklin, Charité - Universitätsmedizin Berlin, Berlin, Germany, e-mail: ulrich.keller@charite.de

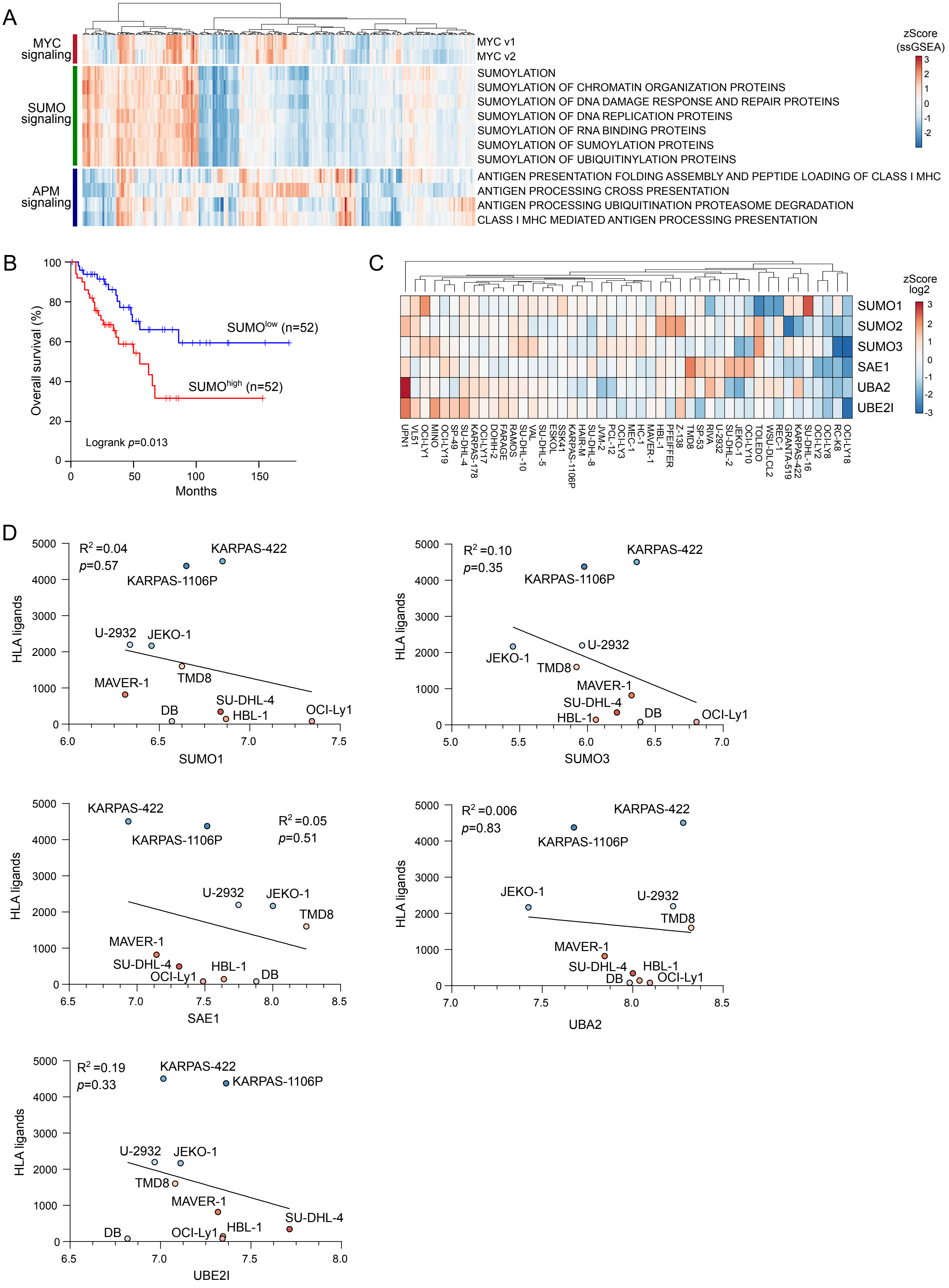

Supplementary Figure S1. (A) Hierarchical clustering (Euclidean/Ward) of ssGSEA scores of normalized human sarcoma transcriptome profiles (TCGA dataset) of indicated MSigDb gene sets belonging to MYC (Hallmark), SUMO (Reactome) and the antigen presentation machinery (reactome). **(B)** Kaplan-Meier-Plot of sarcoma patient dataset (TCGA dataset). The patient survival has been dichotomized in SUMOhigh and SUMOlow based on the expression and subsequent hierarchial clustering (Euclidean/Ward) of the SUMO core machinery. Logrank p-value is indicated. **(C)** Expression of the indicated genes in the human DLBCL cell line dataset (GSE221770). **(D)** Correlation analysis of SUMO1, SUMO3, SAE1, UBA2 and UBE2I expression with the number of unique HLA ligands depicted in Fig.1D.

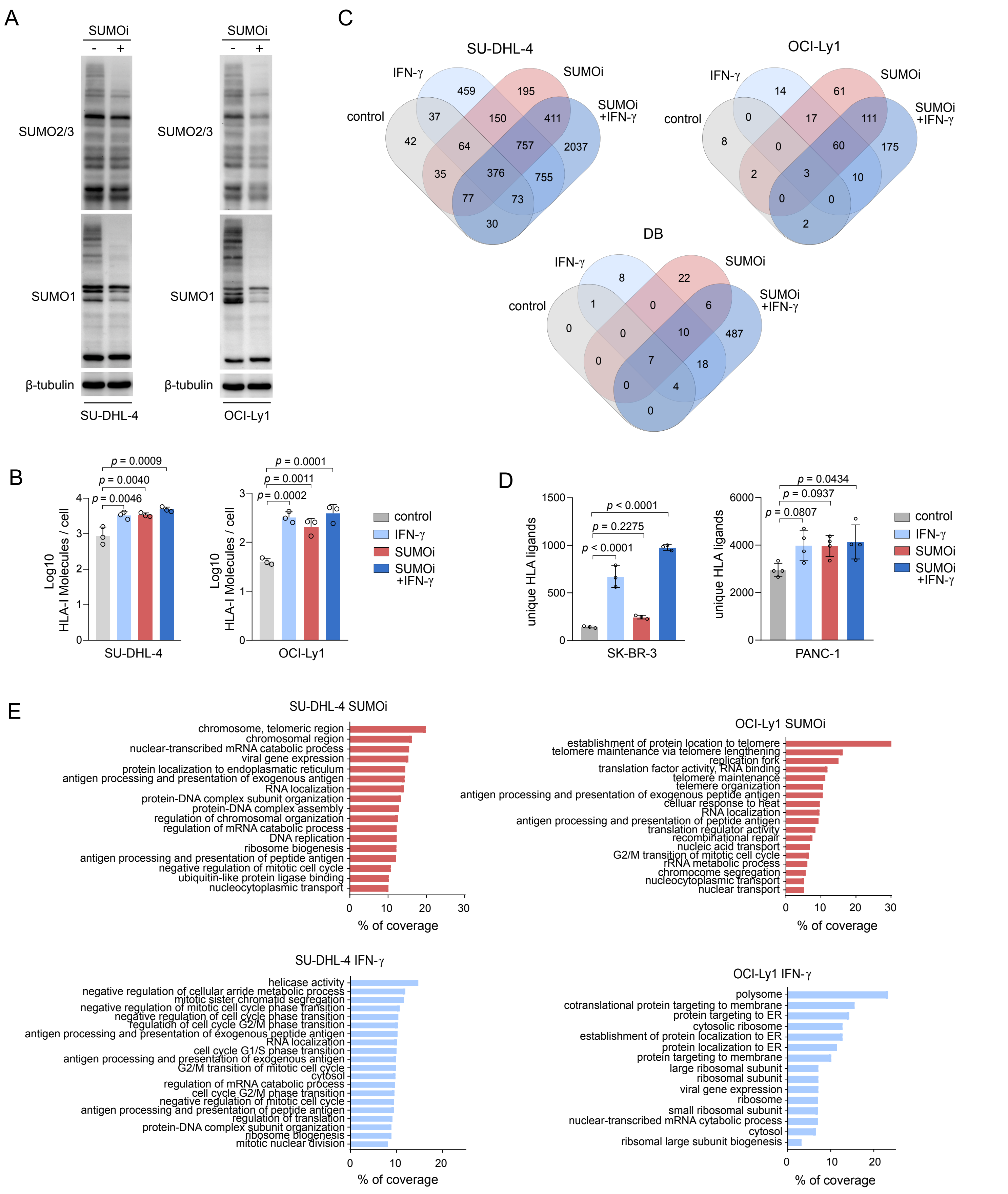

Supplementary Figure S2. (A) Immunoblot analysis of SU-DHL-4 and OCI-Ly1 cells treated with control or SUMOi (400nM, 48h). **(B)** Flow cytometric analysis of the absolute number of HLA-I molecules per cell on SU-DHL-4 and OCI-Ly1 cells treated with control, SUMOi (400nM, 48h), IFN-γ (100U/ml, 24h) or the combination of both (SUMOi: 400nM, 48h; IFN-γ: 100U/ml, 24h). Mean ± SD. ANOVA with Tukey's post hoc test. **(C)** Venn diagrams representing the overlap of the absolute number of all peptides presented on SU-DHL-4, OCI-Ly1 and DB cells in the respective treatment condition. **(D)** Absolute count of unique HLA ligands on SK-BR-3 and PANC-1 cells treated with control, SUMOi (SK-BR-3: 100nM, 72h; PANC-1: 800nM, 72h), IFN-γ (100U/ml, 24h) or the combination of both (SUMOi: SK-BR-3: 100nM, 72h; PANC-1: 800nM, 72h; IFN-γ: 100U/ml, 24h). Mean ± SD. Friedman test. **(E)** Pathway analysis of source proteins of HLA-ligands on SU-DHL-4 and OCI-Ly1 cells, treated with SUMOi (400nM, 48h) or IFN-γ (100U/ml, 24h) using the PantherDB functional annotation tool. Significantly enriched (p<0.05) pathways are depicted.

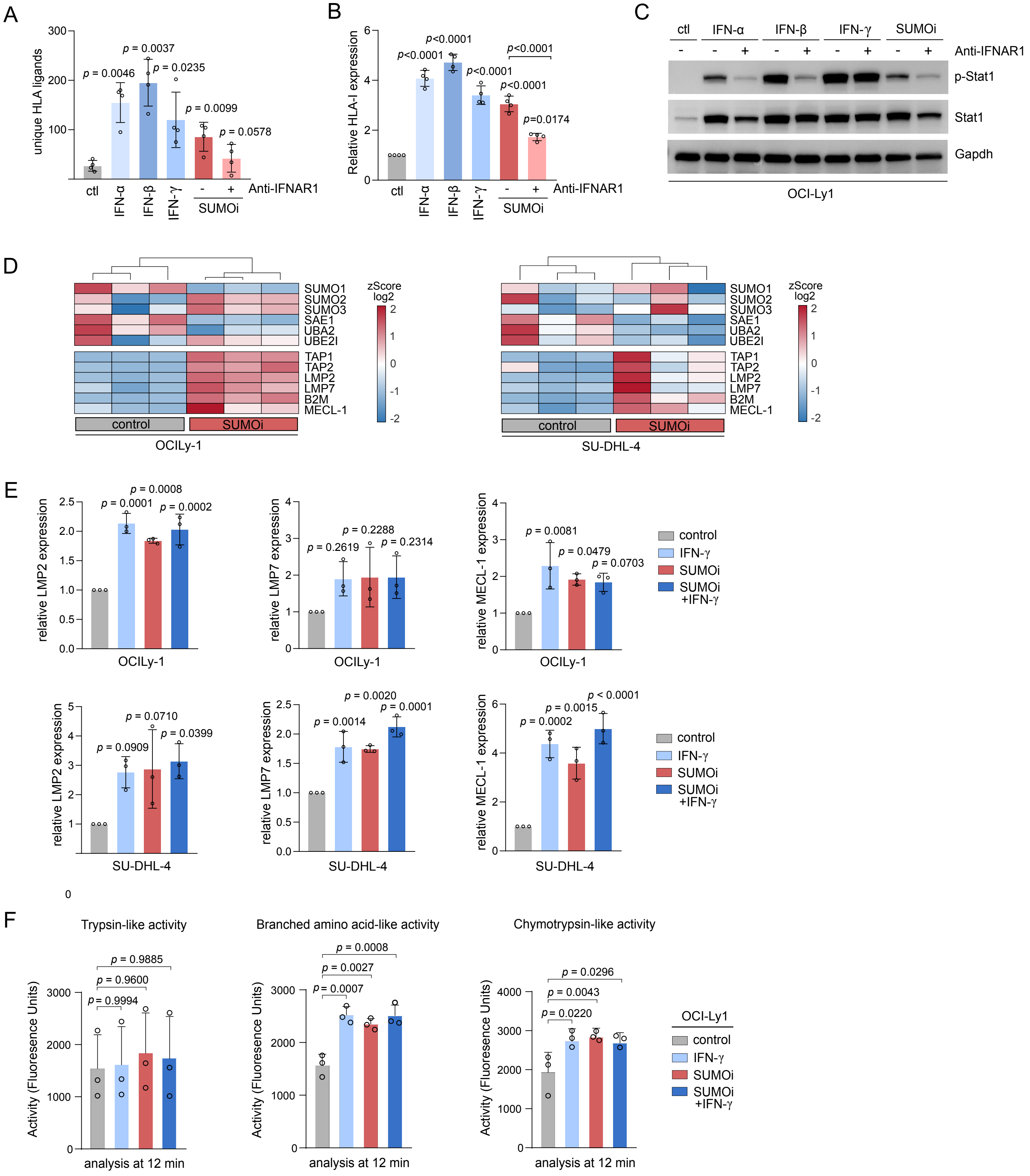

Supplementary Figure S3. (A) Absolute count of unique HLA ligands on OCI-Ly1 treated with control, IFN-α, IFN-β and IFN-γ (all 100U/ml, 24h), SUMOi (400nM, 48h) in presence or absence of anti-IFNAR1 (1000ng/ml, 48h, treatment administration 1h before SUMOi). Mean ± SD. ANOVA with Tukey's post hoc test. **(B)** Relative HLA-I surface expression on OCI-Ly1 cells treated as depicted in (A). Mean ± SD. ANOVA with Tukey's post hoc test. **(C)** Immunoblot analysis of OCI-Ly1 cells treated with control, IFN-α, IFN-β and IFN-γ (all 100U/ml, 24h), SUMOi (400nM, 48h), all in presence or absence of anti-IFNAR1 (1000ng/ml, 48h). **(D)** Expression of the indicated genes in the OCI-Ly1 and SU-DHL-4 cells treated with control and SUMOi (400nM, 48h). **(E)** Quantification of immunoblot analysis of indicated genes in OCI-Ly1 and SU-DHL-4 cells as depicted in Fig. 3A, normalized to β-tubulin. Data represent the mean ± SD. P-values were determined by ANOVA with Tukey's post hoc test. **(F)** Analysis of the immunoproteasome activity in OCI-Ly1 cells treated as depicted in (A) at the 12min timepoint. Data represent the mean ± SD. P-values were determined by ANOVA with Tukey's post hoc test.

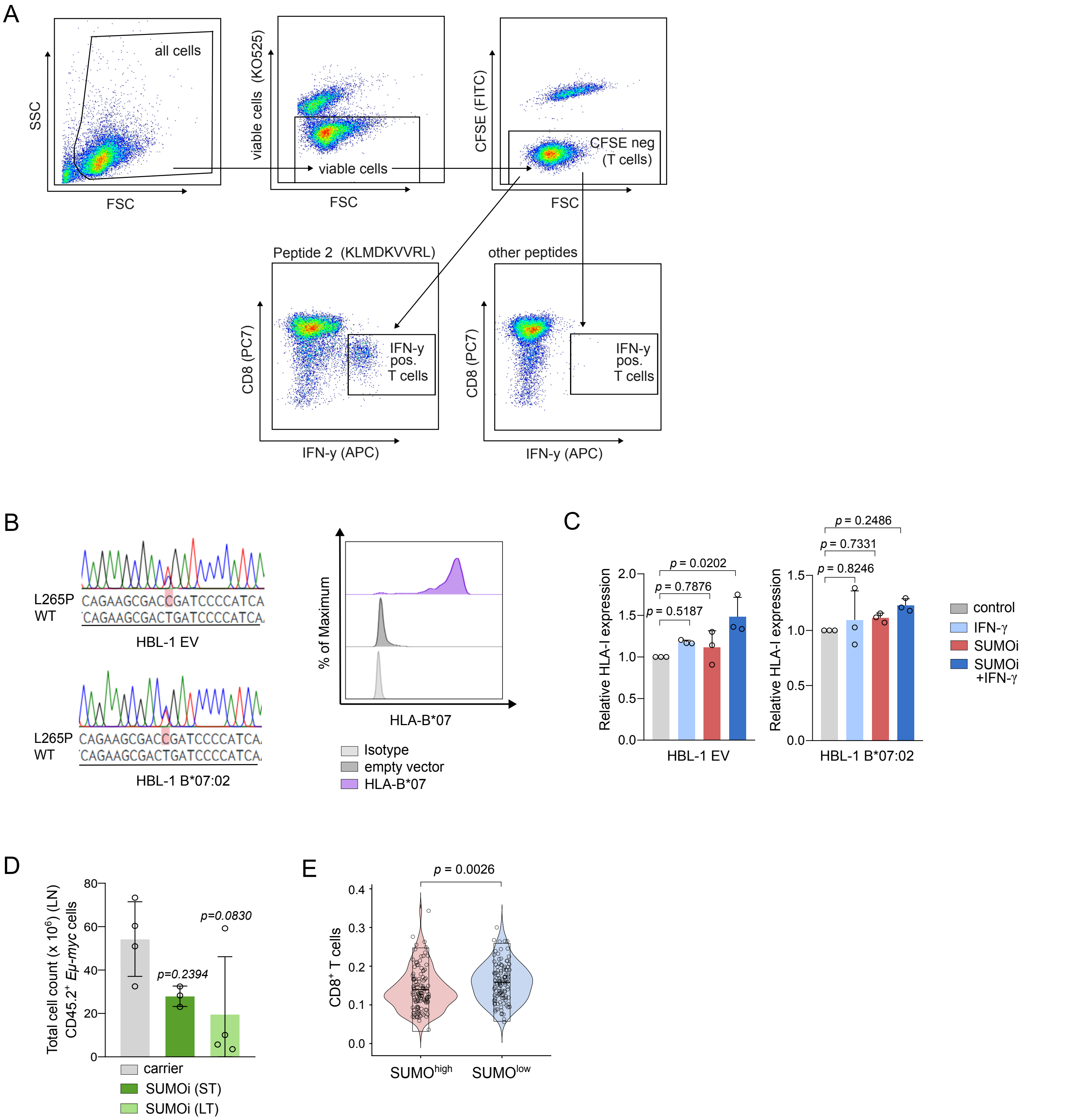

Supplementary Figure S4. (A) Schematic illustration of gating scheme for analysis of IFN-γ production in peptide-primed T cells (KO525 negativ, CFSE negative). **(B)** Schematic illustration of sanger sequencing data of PCR amplified MyD88 L265P vs wild-type (WT) in HBL-1 cells transduced with empty vector (EV) or HLA-B*07:02, combined with flow cytometric analysis of HLA-B*07:02 expression on the respective cell populations. **(C)** Flow cytometric analysis of HLA-I expression on HBL-1 cells transduced with EV or HLA-B*07:02, treated with control, SUMOi (40nM, 48h), IFN-γ (100U/ml, 24h) or the combination of both (SUMOi: 40nM, 48h; IFN-γ: 100U/ml, 24h). Mean ± SD. ANOVA with Tukey's post hoc test. **(D)** Absolute count of Eμ-myc cells (lymph node, LN). Mean ± SD, ANOVA Tukey's post hoc test. **(E)** Analysis of tumor-infiltrating CD8+ T cells with CIBERSORTx (Newman et al. 2015) in the dataset of Hummel et al 2006 clustering in a SUMO-high and SUMO-low subgroup.

SUPPLEMENTAL INFORMATION – Material

Westernblot Antibodies

Target	Dilution	Source	Identifier
β-Actin	1:5000	Sigma-Aldrich	A1978
β-Tubulin	1:5000	DSHB	E7
LMP2	1:1000	abcam	Ab242061
LMP7	1:1000	CST	13635S
MECL-1	1:1000	CST	17579S
TAP1	1:1000	CST	12341S

Reagents and Resources for MS-based proteome analysis

Reagent or Resource	Source	Catalogue number
CHAPS	Sigma-Aldrich	C3023
cOmplete protease inhibitor	Sigma-Aldrich	11836145001
CNBr activated Sepharose	Sigma-Aldrich	C1942
W6/32 antibody	BioXCell	BE0079
tC18 SepPak 50 mg	Waters	WAT054960
Acetonitrile	Merck	1000291000
Trifluoroaceteic acid	ThermoFisher Sc.	85183
MS-grade water	FisherScientific	10505904

Other Reagents and Resources

Reagent or Resource	Source	Identifier
Immunoproteasome activity fluorometric assay kit II	UBPBio	J4170

Software

Software	Source	Identifier
Graphpad version 8.0.1	N/A	https://www.graphpad.com/
R Studio, version 2021.09.2	RStudio: Integrated Development	https://www.rstudio.com

Experimental models: Cell Lines

Cell Line	Source	Identifier
Human: OCI-Ly1	DSMZ	DSMZ no.: ACC 722<br>RRID:CVCL_1879

Human: SU-DH-4	DSMZ	DSMZ no.: ACC 495<br>RRID:CVCL_0539
Human: HBL-1	Cells were kindly supplied by the group of Prof. B. Chapuy, Charité – Universitätsmedizin Berlin<br>RRID:CVCL_4213	
Human: DB	DSMZ	DSMZ no: ACC 539<br>CVCL_1168
Human: MAVER-1	DSMZ	DSMZ no: ACC 717<br>RRID:CVCL_1831
Human: TMD8	Cells were kindly supplied by the group of Dr. A. Busse, Charité – Universitätsmedizin Berlin and Max-Delbrück-Center for Molecular Medizin, Berlin<br>RRID:CVCL_A442	
Human: U-2932	DSMZ	DSMZ no: ACC 633<br>RRID:CVCL_1896
Human: JEKO-1	DSMZ	DSMZ no: ACC 553<br>RRID:CVCL_1865
Human: KARPAS-422	Merck	06101702<br>RRID:CVCL_1865
Human: KARPAS-1006P	Merck	6072607<br>RRID:CVCL_1821
Human: HEK-293	DSMZ	DSMZ no: ACC 305
Human: PANC-1	DSMZ	DSMZ no: ACC 783
Human: SK-BR-3	ATCC	ATCC no: HTB-30

FACS Antibodies

Epitope	Reactivity	Flurochrome	Company	Catalogue number
MHC-I (HLA-A, B, C)	human	APC	Biolegend	311410
IgG2a,k Isotype	mouse	APC	Biolegend	400220
IFNy	human	APC	BD Bioscience	562017
CD8	human	PECy7	Biolegend	344711
HLA-B*07	human	PE	Biolegend	372403
IgG1k Isotype	mouse	PE	Biolegend	981804
MHC-I (H-2kB)	mouse	APC	Biolegend	114714
CD3	murine	PE	Biolegend	100308
CD8	murine	BV786	Biolegend	100750

Spectral Flow Cytometry Antibodies

Epitope	Reactivity	Flurochrome	Company	Catalogue number
CD11c	murine	BUV496	BD	750483
CD44	murine	BUv563	BD	741227
Ter119	murine	BUV615	BD	751534
CD86	murine	BUV661	BD	741502
CD62l	murine	BUV737	BD	612833
MHCII	murine	BUV805	BD	748844
CD45.1	murine	BV421	BioLegend	110732
F4/80	murine	SB436	ThermoFisher	62-4801-80
CD206	murine	PB	BioRad	MCA2235PB
Ly6G/GR1	murine	BV480	BD	746448
CD69	murine	BV510	Biolegend	104531
CD3	murine	BV570	BioLegend	100225
CD11b	murine	BV605	BioLegend	101237
CD23	murine	BV650	BD	740456
CD117	murine	BV711	BioLegend	105835
CD80	murine	BV750	BD	747436
CD138	murine	BV785	Biolegend	142534
H2kB	murine	FITC	BioLegend	116506
CD45.2	murine	RB545	Bd Bioscience	756290
Nk1.1/CD161	murine	PerCP	BioLegend	108725
CD93	murine	BB700	BD	742187
SiglecH	murine	PerCP-efluor 710	ThermoFisher	46-0333-82
PD1	murine	RB780	BD	755860
CD25	murine	PE	Biolegend	113703
CD73	murine	PE/Dazzle 594	BioLegend	127233
CD4	murine	PEfire640	BioLegend	100481
IgM	murine	PeCy5	BioLegend	406544
CD8a	murine	PE-Fire700	BioLegend	100792
Ly6C	murine	PE-Cy7	BioLegend	128018
B220/CD45R	murine	PE-Fire810	BioLegend	103287
CD163	murine	APC	Biolegend	155305
TCRab	murine	AF647	BioLegend	109218
CD19	murine	SPARK-NIR	BioLegend	115568
TCRgd	murine	R718	BD	751919
LD	murine	Zombie NIR	BioLegend	423106
Sca1	murine	APC-Cy7	BD	560654
